# The complete chloroplast genome of *Aloe vera* from China as a Chinese herb

**DOI:** 10.1080/23802359.2020.1726229

**Published:** 2020-02-07

**Authors:** Jia-Ju Ren, Jian Wang, Kai-Ki Lee, Hui Deng, Han Xue, Nan Zhang, Jing-Chun Zhao, Tianyi Cao, Chang-Lei Cui, Xiu-Hang Zhang

**Affiliations:** a School of Nursing, Beijing University of Chinese Medicine, Beijing, China;; b Department of Burn Surgery,The First Hospital of Jilin University, Chaoyang District, Changchun City, Jilin Province, Xinmin Street, China;; c The Chinese University of Hong Kong, School of Preclinicial Medicine, Beijing University of Chinese Medicine, Beijing, China;; d School of Nursing, Beijing University of Chinese Medicine, Fangshan District, Beijing, China;; e Department of Burn Surgery, The First Hospital of Jilin University, Changchun, Jilin, China;; f Department of Clinical Medicine, Zhejiang Chinese Medical University, Hangzhou, Zhejiang, China;; g Department of Anesthesiology, The First Hospital of Jilin University, Changchun, Jilin, China

**Keywords:** *Aloe vera*, *Aloe*, Asphodelaceae, chloroplast, genome

## Abstract

*Aloe vera* has been used as a Chinese herb and an ingredient in many cosmetic products in China. In this study, the complete chloroplast genome of *A. vera* was determined for more genetic data information. The chloroplast genome was 152,875 bp length as a typical quadripartite structure that contained a large single-copy region (LSC) of 83,505 bp, a small single-copy region (SSC) of 16,178 bp and a pair of inverted-repeat regions (IRs) of 26,596 bp. The overall nucleotide composition of chloroplast genome is: 47,185 bp A (30.8%), 48,123 bp T (31.5%), 29,326 bp C (19.2%), 28,241 bp G (18.5%) and the total G + C content of 37.7%. Then, 131 genes were found that included 85 protein-coding genes (PCGs), 38 transfer RNA (tRNAs) and 8 ribosome RNA (rRNAs). The phylogenetic analysis showed that *A. vera* closely related to *A. maculata* in the phylogenetic relationship of the family Asphodelaceae by the Maximum-Likelihood (ML) method.

*Aloe vera* is a perennial, succulent, drought-resistant plant and has been used as a Chinese herb and an ingredient in many cosmetic products in the world, which is belong to the genus *Aloe* and the family Asphodelaceae (Fox et al. 2014). *Aloe vera* contains numerous active ingredients including anthraquinones, polysaccharides, alkylbenzenes, dehydrabietic acid derivatives, salicylic acid, lectin, carotenoids, lignin and saponins so on that attribute for its high therapeutic value (Wynn 2005). Many of the medicinal properties of *A. vera* are ascribed to secondary metabolites, but they are relatively minor in their concentration in the *A. vera* (Dixon 2001). Most of all, *Aloe vera* has been used in Chinese herb for the treatment of a variety of wounds, including skin repair, cold injury, burn injury and pressure ulcers. Nevertheless, less information of *A. vera* about genome dates and studies were been released, and only same information of transcriptome was been released (Pragati et al. 2018). In this study, we had been determined the chloroplast genome of *A. vera* that can be useful for plant genomic studies and phylogenetic analyses, also can use to provide basic data and information for the development of Chinese herb for people in the future.

The Plant Tissues Genomic DNA Extraction Kit (TIANGEN, BJ and CN) was used to isolate the chloroplast genome DNA of *A. vera* and the fresh samples were collected from herb market near Beijing University of Chinese Medicine that located at Fangshan district, Beijing, China (116.17E, 39.71 N). The chloroplast genome DNA was stored in the Beijing University of Chinese Medicine (No. BJUCM-01). Then, the chloroplast genome DNA was purified and sequenced that the raw sequences were quality controlled and removed by the FastQC (Andrews 2015). The chloroplast genome of *A. vera* was assembled and annotated using MitoZ (Meng et al. 2019). The chloroplast genome map of *A. vera* was generated by the OrganellarGenomeDRAW (Lohse et al. 2013). The chloroplast genome sequence of *A. vera* was submitted to GenBank and the accession was No. KX3775242.

The chloroplast genome sequence of *A. vera* was 152,875 base pairs (bp) as a typical quadripartite structure that contained a large single-copy region (LSC) of 83,505 bp, a small single-copy region (SSC) of 16,178 bp and a pair of inverted repeat regions (IRs) of 26,596 bp. The overall nucleotide composition of chloroplast genome is: 47,185 bp A (30.8%), 48,123 bp T (31.5%), 29,326 bp C (19.2%), 28,241 bp G (18.5%) and the total G + C content of 37.7%. The chloroplast genome of *A. vera* contains 131 genes, includes 85 protein-coding genes (PCG), 38 transfer RNA genes (tRNAs), and 8 ribosomal RNA genes (rRNAs).

To study the phylogenetic relationship of *A. vera* with other 12 species chloroplast genomes, we used the maximum-likelihood (ML) method to construct the phylogenetic tree. The phylogenetic tree of ML analysis was performed using the MEGA X (Kumar et al. 2018) with the best model and all of the nodes were inferred with strong support and used the bootstrap values from 2000 replicates. The phylogenetic tree was drawn using the MEGA X and edited using the Evolview web (Subramanian et al. 2019). The phylogenetic analysis of 13 chloroplast genomes showed that *A. vera* is closely clustered with *A. maculata* in the family Asphodelaceae (Figure 1). This study can be useful for plant genomic studies, phylogenetic analyses, and Chinese herb research and development studies of family Asphodelaceae in the future.

**Figure 1.**
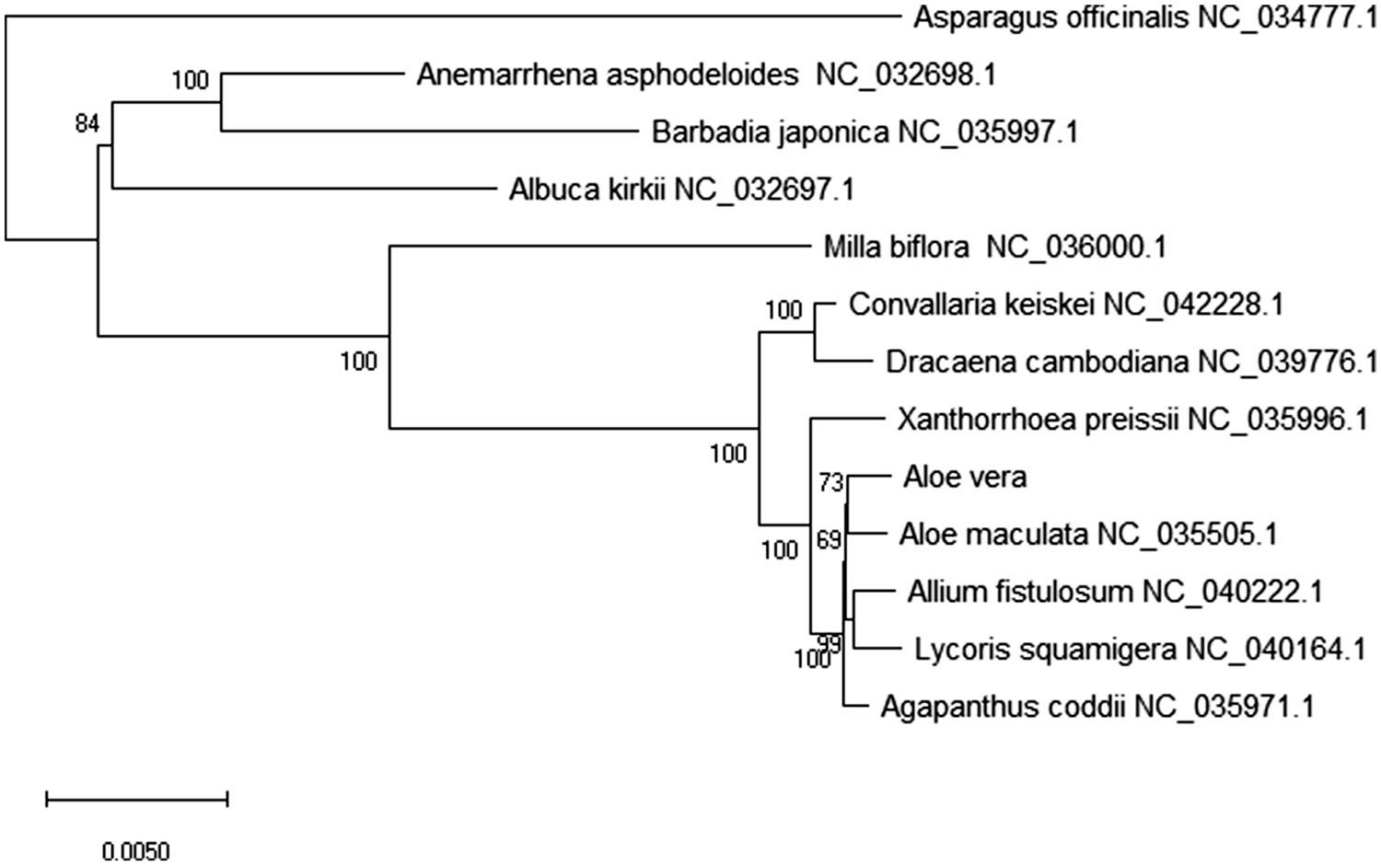
Phylogenetic relationships and analysis of *Aloe vera* with other 12 species chloroplast genomes. Bootstrap support values were given at the nodes and the bootstrap values from 2,000 replicates by the maximum-likelihood (ML) method.
